# Long-Read Sequencing Improves the Detection of Structural Variations Impacting Complex Non-Coding Elements of the Genome

**DOI:** 10.3390/ijms22042060

**Published:** 2021-02-19

**Authors:** Ghausia Begum, Ammar Albanna, Asma Bankapur, Nasna Nassir, Richa Tambi, Bakhrom K. Berdiev, Hosneara Akter, Noushad Karuvantevida, Barbara Kellam, Deena Alhashmi, Wilson W. L. Sung, Bhooma Thiruvahindrapuram, Alawi Alsheikh-Ali, Stephen W. Scherer, Mohammed Uddin

**Affiliations:** 1 College of Medicine, Mohammed Bin Rashid University of Medicine and Health Sciences, Dubai 505055, United Arab Emirates; ghausia.begum@mbru.ac.ae (G.B.); aalbanna@ajch.ae (A.A.); asma.bankapur@mbru.ac.ae (A.B.); nasna.nassir@mbru.ac.ae (N.N.); richa.naveed@mbru.ac.ae (R.T.); bakhrom.berdiev@mbru.ac.ae (B.K.B.); Noushad.Karuvantevida@mbru.ac.ae (N.K.); deena.alhashmi@mbru.ac.ae (D.A.); alawi.alsheikhali@mbru.ac.ae (A.A.-A.); 2 Department of Psychiatry, Al Jalila Children’s Specialty Hospital, Dubai 7662, United Arab Emirates; 3 Genetics and Genomic Medicine Centre, NeuroGen Children’s Healthcare, Dhaka 1205, Bangladesh; hosneara@neurogenbd.com; 4 Department of Biochemistry and Molecular Biology, Dhaka University, Dhaka 1000, Bangladesh; 5 Department of Biotechnology, Bharathidasan University, Tiruchirappalli 620024, India; 6 The Centre for Applied Genomics, The Hospital for Sick Children, Toronto, ON M5S 1A1, Canada; bkellam@sickkids.ca (B.K.); wilson.sung@sickkids.ca (W.W.L.S.); bthiruv@sickkids.ca (B.T.); 7 Department of Genetics and Genome Biology, The Hospital for Sick Children, Toronto, ON M5G 1X8, Canada; 8 McLaughlin Centre and Department of Molecular Genetics, University of Toronto, Toronto, ON M5S, Canada

**Keywords:** long-read sequencing, non-coding RNA, structural variation, whole-genome sequencing (WGS), Oxford Nanopore Technology (ONT)

## Abstract

The advent of long-read sequencing offers a new assessment method of detecting genomic structural variation (SV) in numerous rare genetic diseases. For autism spectrum disorders (ASD) cases where pathogenic variants fail to be found in the protein-coding genic regions along chromosomes, we proposed a scalable workflow to characterize the risk factor of SVs impacting non-coding elements of the genome. We applied whole-genome sequencing on an Emirati family having three children with ASD using long and short-read sequencing technology. A series of analytical pipelines were established to identify a set of SVs with high sensitivity and specificity. At 15-fold coverage, we observed that long-read sequencing technology (987 variants) detected a significantly higher number of SVs when compared to variants detected using short-read technology (509 variants) (*p*-value < 1.1020 × 10^−57^). Further comparison showed 97.9% of long-read sequencing variants were spanning within the 1–100 kb size range (*p*-value < 9.080 × 10^−67^) and impacting over 5000 genes. Moreover, long-read variants detected 604 non-coding RNAs (*p*-value < 9.02 × 10^−9^), comprising 58% microRNA, 31.9% lncRNA, and 9.1% snoRNA. Even at low coverage, long-read sequencing has shown to be a reliable technology in detecting SVs impacting complex elements of the genome.

## 1. Introduction

Structural genomic variants (SVs) such as copy-number variations (CNVs) (deletions and duplications), inversions, and rearrangements account for a large portion of genetic diversity among individuals [1]. In the last two decades, SVs were found to be strongly associated with numerous medical conditions, and notably in neurodevelopmental disorders [2] such as autism [3], schizophrenia [4], and epilepsy [5,6]. Short-read based next-generation sequencing technologies aid in the construction of complex genome assemblies, as well as identification and annotation of critical SVs [5]. However, they require substantial improvement due to the complexity of the genomic context containing significant repeated regions [7,8], thus highlighting the need for more sensitive and accurate technologies to detect and interpret the SVs [9].

Short-read sequencing (SRS) technologies (i.e., Illumina) and their analytical tools are mature, reliable, and cost-effective to detect single nucleotide variants and small indels, however, the sensitivity and specificity to detect SVs across different size spectrums is still rudimentary, specifically within SVs within the complex genomic regions [10]. Studies show that SRS yields many false positives and misconstrues complex rearrangements [11], possibly due to short, amplified reads being unable to reconstruct the original sequence that is greater than the size of the reads [12]. In contrast, long-read sequencing (LRS), i.e., Oxford Nanopore Technologies (ONT) allow ultra-long-reads from 10 kb to 2 Mb [11,13]. ONT uses molecularly engineered nanopores to sequence long-reads of DNA in real-time using portable and low-cost sequencers [14]. Research shows that nanopore technology builds high-quality reference genomes [15], de novo assembly [16], and fills the gaps missed by SRS, thus, enabling easy characterization of SVs and discovery of novel variants [17].

Moreover, numerous tools and databases have been developed to identify the role of SVs in the pathogenesis of diseases by essentially focusing on the functional units (i.e., exons) of the genome [18]. However, for decades, SVs affecting non-coding elements of the genome, such as microRNAs (miRNA), long non-coding RNAs (lncRNA), and other non-coding elements, were neglected and remain poorly understood as they were considered transcriptionally silent [19,20]. Emerging studies have highlighted the role of functionally significant elements in non-coding regions of the genome and their contribution to diseases, such as cancer [21], neurodevelopmental disorders, such as autism [22], and neurodegenerative diseases, such as Alzheimer’s disease [20].

In this study, we examined a multi-generational Emirati family with triplets (MZ twins and DZ triplets) impacted by autism spectrum disorders (ASD). Clinical exome testing reported negative for pathogenic variants associated with autism in protein-coding genes. In attempt to find novel SVs contributing to the etiology of ASD in this family, we therefore designed a workflow to explore the effect of such genetic variation in the underrepresented complex non-coding elements into the risk factor of autism spectrum disorders. The application of this study will be critical to increase the genetic diagnostic yield in the future for autism cases with negative genetic tests from clinical exome sequencing. To facilitate these aims, we are comparing the two whole-genome sequencing (WGS) platforms—SRS and LRS technology for the characterization of SVs detection sensitivity and specificity.

## 2. Results

A complex nuclear family with triplets (monozygotic and dizygotic siblings) impacted by ASD was initially tested using clinical microarray and whole-exome sequencing. The analysis did not yield any obvious pathogenic variants. We have conducted whole-genome sequencing using illumina technology and identified a variant of unknown significance (VOUS) 151 kb deletion impacting the *GOLGA8B* gene in chromosome 15 in the triplets (MZ twins and the dizygotic twin). Our analysis of de novo SNVs identified variants impacting *DXO* and *CLCA4* gene were classified a VOUS following the American College of Medical Genetics and Genomics (ACMG) guidelines (Figure S1).

**Figure 1:**
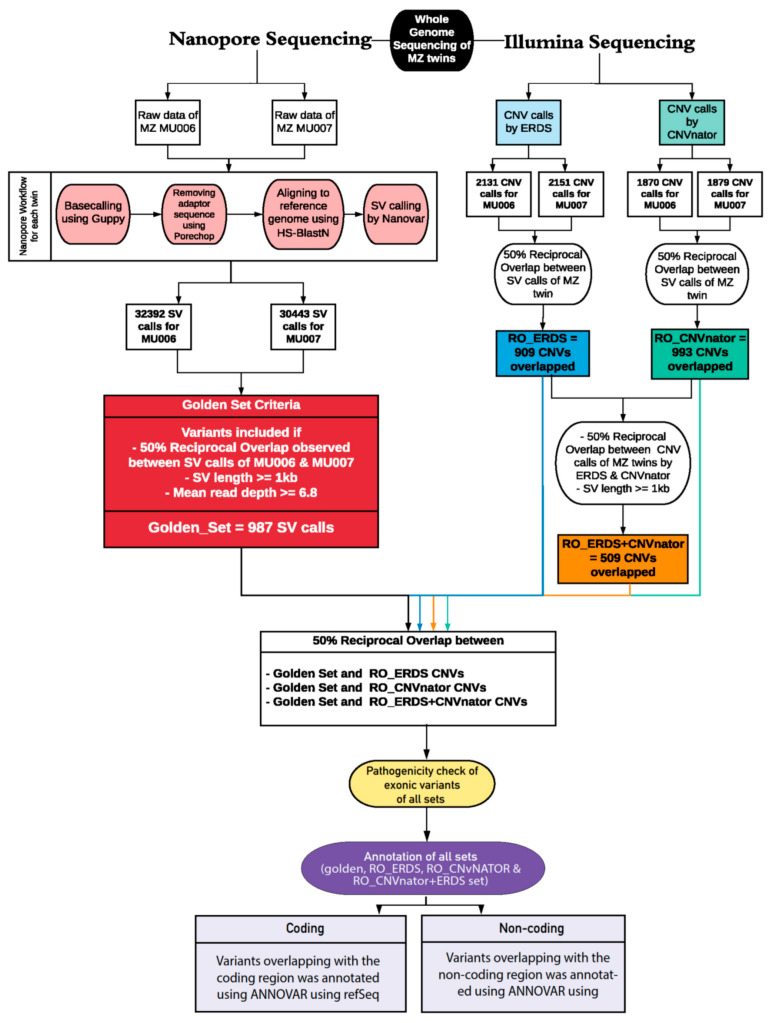
Overview of the Structural Variant Calling workflow for whole-genome sequencing data performed by two technologies—long-read sequencing by nanopore sequencing and short-read sequencing by llumina sequencing—for detection of structural variants in monozygotic twins (MU006 and MU007). For the first analysis, the raw fast5 files acquired from Oxford Nanopore Technologies (ONT) sequencer were processed using the following steps for each monozygotic twin: base calling done by Guppy, filtering by Porechop, mapping to hg38 reference genome using HS-BlastN, and Nanovar was used for calling structural variant. For the second analysis, the CNV calls from Illumina sequencing were established using two callers: CNVnator and ERDS caller. To determine identical variants between the twins for each caller—Nanovar, CNVnator, and ERDS—we performed a 50% reciprocal overlap analysis on their VCF outputs and filtered out variants below 1 kb to formulate the ‘golden set’, RO_CNVnator set, and RO_ERDS sets, respectively. To investigate identical variants between the LRS and SRS platforms, reciprocal over-lap analysis was done on the ‘golden set’, RO_CNVnator, and RO_ERDS sets. All sets were investigated for pathogenicity and annotated using Annovar to determine the coding and non-coding genomic elements overlapping with the examined variants.

In such a scenario, where clinically diagnosed patients exhibited a low likelihood of pathogenic variants contributing to their condition in the protein-coding genes, we aimed to study the underrepresented complex non-coding elements of the genome. Previous literature has suggested long-read sequencing could identify functionally relevant non-coding elements in neuropsychiatric disorders [23] and cancer [24]. Therefore, we designed an analytical pipeline to detect and characterize a comprehensive set of SVs (Figure 1).

**Figure 2:**
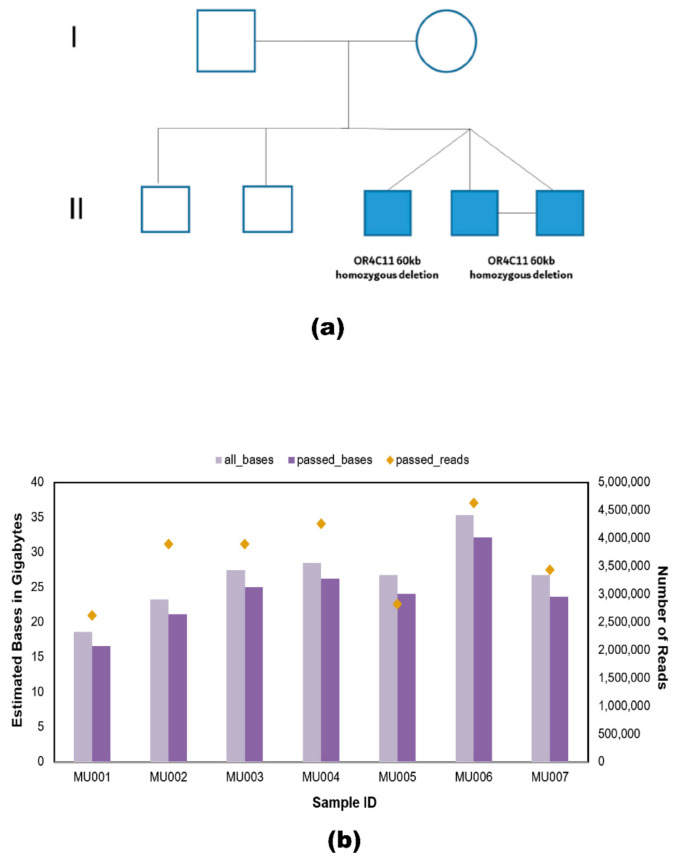
Pedigree and summary of all samples sequenced by nanopore sequencing. (**a**) Schematic representation of the pedigree affected by autism spectrum disorder exhibiting variants of unknown significance. Affected triplets are represented in shaded squares originating from one point and MZ twins (MU006 and MU007) are indicated with a horizontal line joining their shaded squares. Pedigree displays a 60 kb de novo deletion in the *OR4C11* gene present in the MZ twins and the dizygotic twin. (**b**) Representation of the whole-genome sequenced data per sample (parents, unaffected brothers, and the triplets) in terms of the number of bases sequenced using the MinION sequencer. All bases represented in light purple are unfiltered and the passed bases depicted in dark purple have been filtered to remove low-quality reads. All passed reads possess a quality score of 7 or above. The estimated bases are represented in gigabytes. The secondary y-axis indicates the number of passed reads generated. Yellow diamonds represent the number of passed reads per sample. This data was formulated using pycoQC v2.5.0.23.

The long-read throughput of each family member varied due to differences in sequencing run time, and the stochastic fluctuation of open pores in flow cells. A schematic representation of the family’s pedigree affected by autism spectrum disorder is depicted in Figure 2a. Basecalling was performed using Guppy for all samples and data from individual flow cells were combined, which produced 29,623,679 reads out of which 25,537,921 were passed reads having the minimum quality of 7 or above. The passed reads contained a total of 168.49 Gb of DNA sequence. The highest throughput recorded was for one of the MZ twins (MU006) with a yield of 32.08 Gb of passed bases, and the lowest throughput recorded was for the mother’s sample (MU001) with a yield of 16.58 Gb of passed bases (Figure 2b).

Details of the MinION sequencing summary of all samples can be found in Table S1. The median read length across all samples was 4.02 kb and the longest read length obtained was 113.39 kb. The mean coverage for each sample is 6.8. For the MZ twins (MU006 and MU007), a total of 8,061,176 base called reads passed the quality threshold, generating a combined total of 52 Gb bases with a median Phred score of approximately 10.8 (Figure S2). Alignment to the human reference genome (hg38 build) was achieved with an accuracy of 90.5% of the twin’s sample reads with an average read N50 of 11,650 base pairs and total coverage of 15.59-fold.

**Figure 3:**
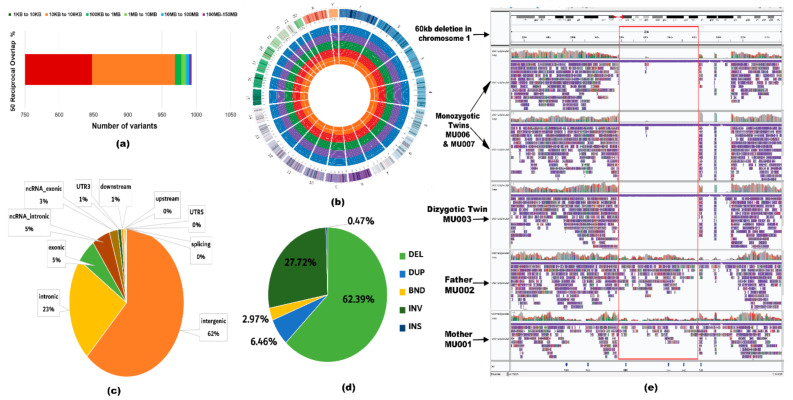
Characteristics of the structural variants (SV) belonging to the ‘golden set’. (**a**) Distribution of SV length in the ‘golden set’ of variants. In the stacked bar chart, each color bar portrays the number of variants belonging to a range of SV lengths spanning from 1 kb to 150 Mb. (**b**) A circos genome map demonstrating variants based on different classes of SVs per chromosome. Considering the concentric circles from the innermost circle to the outermost, the orange circle depicts deletions, the red circle depicts duplications, the green circle depicts inversions, the purple circle depicts insertions, the blue circle depicts the breakends, and the multi-color outermost circle indicates the location of the chromosome. Each dot on the concentric circles represents a variant of the ‘golden set’. (**c**) Identification of the coding and non-coding elements overlapping with the variants represented in a pie chart. (**d**) Evaluating the number of genes overlapping with different types of SVs. Each slice describes a type of SV (deletion, duplication, inversion, insertion, or breakend) and the percentage describes the portion of genes overlapping with that SV type. (**e**) Screenshot of the alignment of the 60 kb de novo deletion detected by LRS sequencing, visualized using Integrative genomics viewer (IGV). From top to bottom, the alignment is as follows: MU007 and MU006 (monozygotic twins), MU003 (dizygotic twin), MU002 (father), and MU001 (mother). The blank lines in the alignment of MU007, MU006, and MU003 show the homozygous deletion in the *OR4C11* gene. It is clear from the alignment of the reads that this deletion is present only in the proband and not in the parents.

We have applied NanoVar, a structural variation caller for long-read sequence. NanoVar utilizes low depth WGS data to call highly accurate SVs [25]. NanoVar generated a comprehensive Variant Call Format (VCF) file of 32,392 and 30,443 SV (including indels) calls for MU006 and MU007, respectively. To exhibit identical SVs between the twins, a 50% reciprocal overlap threshold was applied. These overlapping variants were further filtered in terms of SV length and read depth. Only variants above SV length of 1 kb and read depth of 6.8 or above were included. This overlapping variant set derived from the MZ twins referred to as ‘golden set’ comprising of 987 SV calls. Out of 987 SV calls, the SV length of 967 calls belonged to the size range of 1 to 100 kb (Figure 3a). SVs represented in the ‘golden set’ belonged to the following classes: (643 calls) 65.1% deletion (DEL), (179 calls) 18.1% tandem duplication (DUP), (50 calls) 5% inversion (INV), (17 calls) 1.7% insertion (INS, novel sequence insertion/insertion of sequences absent from reference genome), and (98 calls) 9.9% breakends (BND) (Figure 3b). Gene-based and region-based annotation using ANNOVAR revealed 5.4% of variants affected the exonic region of the genome while 94.6% were in non-coding regions (Figure 3c). We further examined 5360 genes affected by the ‘golden set’ variants (Figure 3d). NanoVar identified a 60 kb de novo deletion in the *OR4C11* gene in chromosome 11 shared by the triplets, as demonstrated in the pedigree (Figure 2a). The alignment of parents and triplets showed a clear homozygous deletion in the latter, as visualized using IGV Viewer (Figure 3e). The variant was also detected by SRS sequencing, further validating the variant, however, the functional consequences of this de novo deletion to ASD are unknown.

**Figure 4:**
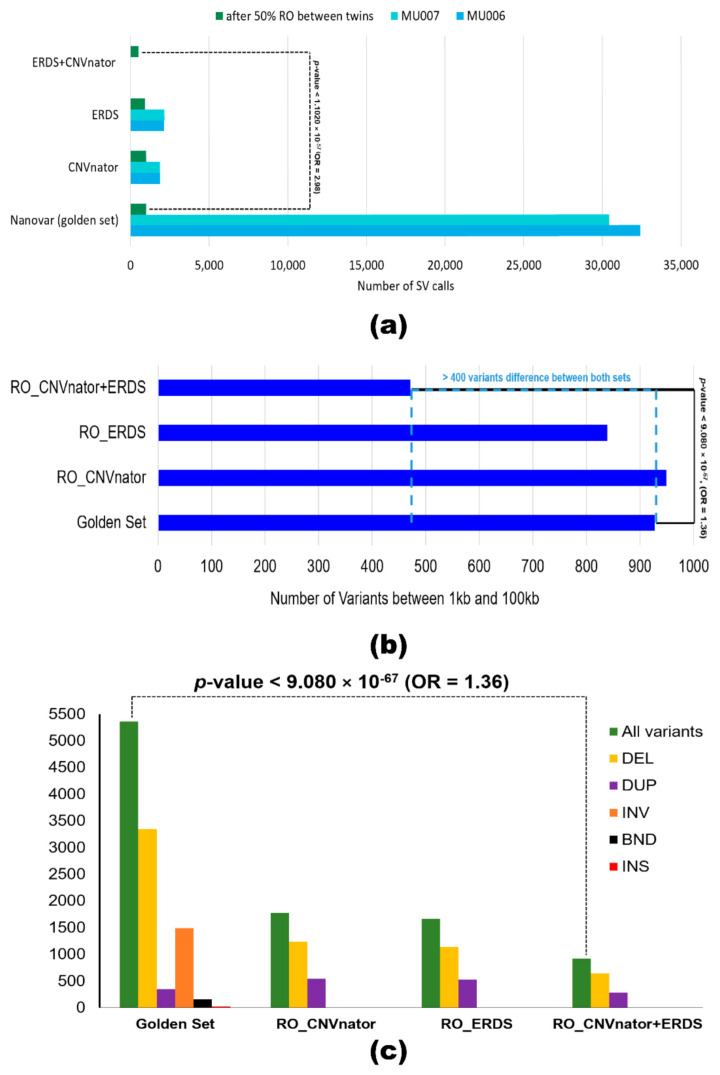
Comparison between variant sets formulated by polishing LRS calls (‘golden set’) and SRS calls (RO_CNVnator, RO_ERDS, and RO_CNVnator + ERDS sets). (**a**) Bar graph representing the number of variants called for each of the monozygotic twins and after their reciprocal overlap analysis by callers—Nanovar (in green), ERDS (light blue), and CNVnator (dark blue). (**b**) Assessment of the number of structural variants present within the 1 to 100 kb size range for each set is represented in a bar graph. (**c**) Comparison between the number of genes overlapping with variants called by the different sets of LRS and SRS callers and a breakdown of the gene distribution per variant type—deletions (in yellow), duplications (in purple), inversion (in orange), breakends (in black) and insertions (in red). The *p*-value and odds ratio show the association between long-read caller NanoVar and its ability to call a number of variants (**a**) within the 1–100 kb size range, (**b**) affecting the number of genes, and (**c**) when compared to illumina consensus set.

To achieve a standardized framework for comparison, we optimized and annotated the SRS calls using the same parameters as the ‘golden set’. ERDS and CNVnator callers were used for variant calling. RO_ERDS set and RO_CNVnator set were generated by finding a 50% reciprocal overlap between the MZ twins resulting in 993 CNVs and 907 CNV calls, respectively (Figure 4a). To achieve better accuracy, an ‘illumina consensus set’ (RO_ERDS_CNVnator) was produced, which contained 502 CNV calls identical to the MZ twins across both callers. For all three approaches, the majority (>74%) of the variants were recorded to be deletions, while the rest were duplications. The analysis showed 91.3% of variants belonged to the long 1–100 kb range (Figure 4b), of which 13.7% of variants were overlapping with the protein-coding region and 924 genes were impacted by the ‘illumina consensus set’ of variants (Figure 4c). Associations between ‘golden set’ and number of variants identified (*p*-value = 1.1020 × 10^−57^; OR = 2.98), long variants (*p*-value < 9.080 × 10^−67^; OR = 1.36), and number of genes impacted (*p*-value < 9.018379 × 10^−9^; OR = 21.04) compared to the ‘illumina consensus set’ of RO_CNVnator + ERDS SVs were statistically significant.

To identify shared variants between both platforms (SRS and LRS), reciprocal overlap with a threshold of 50% [1] was performed between the following sets: ‘Golden set’ and RO_ERDS set, ‘golden set’ and RO_CNVnator set, and ‘golden set’ and RO_ERDS_CNVnator set, which resulted in 473, 314, and 284 SV calls overlapping between sets, respectively (Figure S3). Variants from all sets were examined for pathogenicity. We looked for variants classified as de novo or inherited pathogenic, likely pathogenic, benign, and VOUS. The pathogenicity assessment revealed a 60 kb de novo deletion (Figure 3e) in the *OR4C11* gene to be benign.

**Figure 5:**
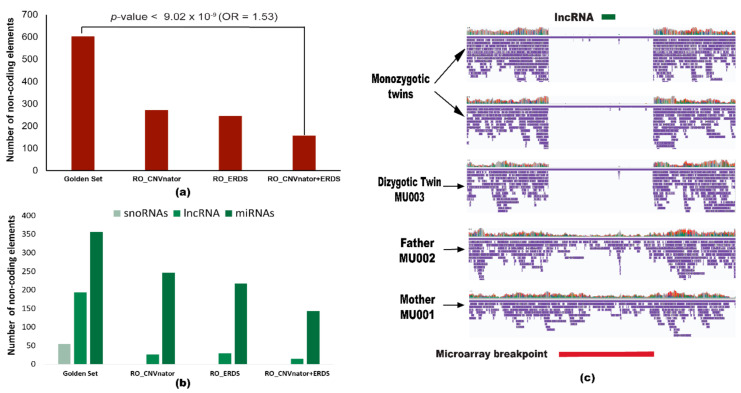
Prevalence of non-coding elements among structural variants. (**a**) Detection of all non-coding RNAs overlapping with different sets of LRS and SRS callers. The *p*-value and odds ratio denote the ability of long-read caller NanoVar (‘golden set’) to call variants overlapping with the non-coding elements compared to illumina consensus set (RO_CNVnator + ERDS). (**b**) Bar plots demonstrate the frequency of a type of non-coding RNA (small nucleolar RNA, long non-coding RNA, and microRNA) overlapping with structural variants in each set. (**c**) Screenshot of the alignment of the 122 kb deletion (chr19: 20,412,999–20,535,167) detected in the affected triplets by the golden set visualized using Integrative genomics viewer (IGV). The red bar denotes the microarray breakpoint (chr19: 20,415,010–20,532,427) and the green bar represents the 1.4 kb lncRNA in band 19p12.

We further annotated the variants to understand their impact on the non-coding elements of the genome. Annotation of the ‘golden set’ revealed that 94.6% of the variants overlapped with the non-coding elements of the genome, while for SRS callers the highest overlap was of 88.2% and 30.8% for the RO_ERDS and ‘illumina consensus set‘ (RO_CNVnator + ERDS set), respectively. We also analyzed different types of non-coding RNAs identified by these two platforms viz. microRNAs (miRNA), long non-coding RNA (lncRNA), and small nucleolar RNA (snoRNA). The ‘golden set’ overlapped with 604 non-coding RNAs (Figure 5a), out of which there were 356 miRNAs, 193 lncRNAs, and 55 snoRNAs (Figure 5b). None of the SRS sets detected snoRNAs. The maximum number of miRNAs detected by SRS technology was 29 by the RO_ERDS set. While the RO_CNVnator and RO_ERDS set identified 246 and 217 lncRNAs, respectively, the illumina consensus set could verify only 143 lncRNAs. Association between the ‘golden set’ and the number of ncRNAs identified compared to the illumina consensus set was statistically significant (*p*-value < 9.02 × 10^−9^; OR = 1.53). Moreover, out of the 437 calls unique to the ‘golden set’, Nanovar identified a 122 kb de novo deletion in chromosome 19 in the *ZNF826P* gene overlapping with 1.4 kb lncRNA (Figure 5c). This deleted variant was further validated by microarray, and it was missed by the ‘illumina consensus set’.

## 3. Discussion

We report the whole-genome LRS for a family with triplet children diagnosed with ASD. The sequencing of MZ twins with ASD using LRS by nanopore technology produced a significantly higher number of SV calls (987 SV calls) compared to variants sequenced using short-read sequencing technology (509 CNV calls). Most lncRNAs contain transposable elements [7] and due to this sequence complexity, short-read technology is often unable to conduct alignment properly. This eventually led to noisy calls in SVs using SRS. At approximately 15-fold coverage, 49% of SVs (484 putative SV calls) called by NanoVar (LRS caller) were unique to the LRS set and not found in either the CNVnator and ERDS caller (SRS callers). This is consistent with studies that reported long-read reference mapping to have a low coverage requirement (10–15-fold) for SV detection [26] while short-read sequencing captures roughly 85% of the genome, leaving out most of the variation-rich regions [27]. There is substantial evidence that SVs contribute to phenotypic variation and provide insights into the pathogenesis of human diseases [28], thus missing critical SVs by having a high coverage requirement may hinder our understanding of the significance of SVs in disease pathogenesis.

Moreover, challenging subtypes of SV [29,30], insertion of novel sequences (1.7%) and inversions (5%) were uniquely detected by LRS technology and missed by SRS technology. Our findings further revealed that the number of genes spanning SV calls (NanoVar) were almost six times more than the CNV calls detected (CNVnator + ERDS) and approximately three times more if compared to a single short-read caller (CNVnator or ERDS). Out of 5360 genes that were impacted by variants called by NanoVar, 72.3% of the calls were deletions, while 27.7% were inversions. Inversions are known to either be directly associated with mental retardation or increase the risk of further rearrangement [31]. Traditional sequencing methods often fail to detect inversions [29], whereas LRS has reportedly identified large inversions disrupting genes for samples facilitating in preimplantation genetic testing [32]. Our assessment confirms the potential of nanopore technology and the shortcomings of SRS technology in detecting challenging subtypes of SVs, which are overlooked in genomic studies. The cost difference between long-read and short WGS is contracting every year, making the use of LRS more feasible.

We further report that 97.9% of SVs belonged in the long-range (1–100 kb) as detected by nanopore technology, while only 91.1% of variants were discovered by SRS technology. Studies have shown that long-range variants are optimal for haplotyping variants across genes [30]. Thus, the importance of their detection in the human genome is supported by nanopore technology, as observed in our analysis.

In our study, we intended to explore these non-coding elements impacting the SVs and our analysis revealed that the majority of the variants examined using LRS were positioned outside the coding regions. The repetitive sequence complexity of functional non-coding RNA (ncRNA) [7,33], and the continual discovery of new ncRNAs [34], makes it challenging to cover all types of ncRNAs, hence, we opted for miRNA, lncRNA, and snoRNA. Studies have shown that miRNA regulates gene expression in neurodevelopment, and, at a minimum, 11 miRNAs have been reported to be directly associated with ASD [22], and dysregulated miRNA expression has been reported in the brain of Alzheimer’s patients [21]. Studies revealed the role of lncRNAs in immunity, specifically in proliferation, differentiation, and activation of immune cells [35], while their association with CNVs causing ASD is also being studied [36]. Moreover, microdeletion of snoRNA is associated with neurodevelopmental disorders such as Prader–Willi syndrome-like phenotype [37]. This highlights the contribution of these ncRNAs in the etiology of diseases and hence emphasizes the need to investigate how SVs might affect their function.

We additionally report the identification of 604 ncRNAs overlapping with variants called using NanoVar, surpassing the number of ncRNAs called by both individual SRS callers CNVnator (272), ERDS (246) as well as the illumina consensus set (157). Looking further into the distribution of types of ncRNAs, NanoVar variants identified all three ncRNAs of interest while SRS sets revealed only miRNAs and lncRNAs, missing snoRNAs. In addition to that, the detection of miRNAs affected by the Illumina set of variants was 8.1%, while for NanoVar it was 60% of the total variants. Our evaluation depicts a substantial increase in the detection of crucial ncRNAs by LRS sequencing, outdoing short-read sequencing variants. Moreover, as per our study, we recommend the inclusion of the analysis of non-coding elements in genomic studies, which will further help in elucidating and annotating functional SVs.

In our study, we have developed a scalable workflow using LRS by nanopore technology for the detection of SVs impacting both protein-coding and non-coding elements of the genome, which are emerging as important regulators in neurodevelopmental disorders [38]. Even at low coverage, LRS sequencing has proven to be a resourceful and reliable technology in overcoming the inadequacies of short-read sequencing and serves as a promising tool to interrogate different forms of variation associated with human diseases. In the era of WGS, we infer that our workflow will facilitate the analysis and interpretation of SVs in the human genome.

## 4. Materials and Methods

### 4.1. DNA Sample Source

The MZ twins sequenced in this study were recruited from Al Jalila Children’s Hospital, where they were clinically diagnosed with ASD through a comprehensive multidisciplinary clinical assessment using the Diagnostic and Statistical Manual for Mental Disorders—5th Edition (DSM-5) criteria and Autism Diagnostic Observation Schedules, second edition (ADOS-2) administered by research-reliable assessors. The study received approval from the institutional review board of the Mohammed Bin Rashid University of Medicine and Health Sciences. Patients and their family members gave written consent before sample collection. Blood samples were acquired from the MZ twins (MU006 and MU007) and their family members: The dizygotic twin (MU003), two brothers (MU004 and MU005), mother (MU001), and father (MU002). All subjects were from the United Arab Emirates.

### 4.2. Genomic DNA Extraction

Genomic DNA was extracted from blood using the QIAamp DNA Blood Midi Kit (Qiagen, Inc., Valencia, CA, USA) according to the manufacturer’s protocol. The kit uses the silica membrane-based DNA purification method for extracting DNA. After purification using spin columns, 200 μL of DNA was eluted with a total concentration of 3–20 μg. The concentration of all samples was determined using the Qubit 2.0 fluorometer system, and purity evaluation of genomic DNA was done using the Nanodrop spectrophotometer system. The ratio of OD 260/280 and 260/230 of 1.8 and 2 was maintained, respectively, for nanopore and Illumina sequencing.

### 4.3. Library Preparation

#### 4.3.1. Nanopore Sequencing

Sequencing libraries were generated using the PCR- free SQK-LSK109 ligation kit from ONT. The input sample varied from 1.5 to 1.8 ug and DNA LoBind tubes were used for sequencing. Library preparation started with DNA repair/A-tailing using the NEBNext FFPE repair mix (#M6630) and the NEBNext End repair/dA-tailing module (E7546), followed by AMPure XP bead clean-up. Adapter ligation was performed using the NEB T4 ligase (NEBNext Quick Ligation Module, #E6056) and the LSK109 compatible adapter mix (AMX) and ligation buffer, respectively. Samples were again purified using AMPure XP beads, using the Long Fragment Buffer (LFB) for the wash steps. Final sample elution from the beads was done using 15 μL of elution buffer (EB). Samples were quantified using a Qubit fluorimeter and diluted appropriately before loading onto the flow cells.

#### 4.3.2. SRS Using Illumina Sequencing

The genomic DNA extracted from QIAamp DNA Blood Midi Kit (Qiagen, Inc., Valencia, CA, USA) was sent to Center for Applied Genomics (Toronto, ON, Canada) for SRS. Illumina sequencing was done as per [39].

### 4.4. Whole-Genome Sequencing

#### 4.4.1. Nanopore Analytic Pipeline

Following ONT standard operating procedures, samples were loaded onto MinION compatible flow cells. A total of 18 flow cells were used, out of which five were R9.4.1 version and the remaining were RevD flow cells. We also used MinIT mobile sequencing device from ONT to run multiple samples simultaneously without the need for another laptop. Data acquisition varied from 48 to 72 h per run. Average mean coverage of six was observed. The sequencing summary is available in Supplementary Table S1.

##### Basecalling and Mapping

The raw data from the sequencers were basecalled using Guppy (7 individuals, 18 flow cells). We used Guppy Sequencing Pipeline Software (https://github.com/nanoporetech/pyguppyclient (accessed on 15 November 2020)), v 2.3.1, with model configuration file template template_r9.4.1_450bps_5mer_raw_prom.jsn for MinION flow cells. Porechop software (https://github.com/rrwick/Porechop (accessed on 15 November 2020)), v 0.2.3 (https://github.com/rrwick/Porechop (accessed on 15 November 2020)) was used to find and trim the adaptor sequences from our sample reads using the command: *‘Porechop -i input_reads.fastq.gz -o output_reads.fastq.gz’*.

The trimmed base called reads were mapped to human reference genome GRCh38 with HS-BLASTN, (https://github.com/benoukraflab/NanoVar (accessed on 15 November 2020)), v 2.14-r883, using the recommended option for ONT sequence to reference mapping ‘-x map-ont’ and we used the parameters ‘--MD -Y’. The aligned reads were sorted using samtools and stored in a bam file.

##### SV Calling

The sorted aligned reads were called for SVs using NanoVar (https://github.com/benoukraflab/NanoVar (accessed on 15 November 2020)), v 1.3.8. To find overlapping variants between the MZ twins, 50% reciprocal overlap analysis was performed on MU006 and MU007 using Reciprocal Overlap Annotator (Broad Institute). Variants greater or equal to 1 kb in length and mean read depth of 6.8 or above were included.

#### 4.4.2. SV Calls Using SRS

For WGS, the TCAG DNA-Seq pipeline used GATK 3.7 for variant calling. Alignment was performed using bwa-mem algorithm, version 0.7.12, and duplicate reads are marked using Picard Tools, v 2.5.0. Two SRS callers were used in our pipeline—ERDS, version 1.1, and CNVnator, v 0.3.2. Since the reads were mapped to the hg37 reference sequence, we used Liftover, v 1.1.6, to convert the reads to hg38 build. RO_ERDS and RO_CNVnator set were produced after performing 50% reciprocal overlap using bedtools on MZ variants called by ERDS and CNVnator callers, respectively. After excluding variants below 1 kb in length, a 50% reciprocal overlap between RO_ERDS and RO_CNVnator variants was investigated.

### 4.5. Comparing Variants Called by Both Platforms

To find overlapping variants between both platforms, reciprocal overlap between the ‘golden set’ and RO_ERDS set, ‘golden set’ and RO_CNVnator set and ‘golden set’ and RO_ERDS_CNVnator set was established. The resultant overlapping variants were annotated using Annovar (https://annovar.openbioinformatics.org (accessed on 15 November 2020)), v—24 October 2019. To compare the performance of both platforms and to check if there is an association between the type of caller and factors, such as the total number of variants called, long variants, and numbers of genes impacted, Fisher’s exact test was performed using RStudio software (https://cran.r-project.org (accessed on 15 November 2020)), v—1.2.5033.

### 4.6. Overlapping Set and Validation

The overlapping set of variants between both SRS and LRS platform was examined for pathogenicity to identify novel and VOUS variants. Databases such as Database of Genomic Variants (DGV) were used to check the frequency of the variant and ACMG (https://www.acmg.net (accessed on 15 November 2020)), Clinvar (https://www.ncbi.nlm.nih.gov/clinvar (accessed on 15 November 2020)), and OMIM (https://www.omim.org (accessed on 15 November 2020)) databases were utilized to check supporting evidence for pathogenic variants. Commercial whole-exome sequencing was also performed on the family to identify pathogenic variants. Validation of variants was performed using Sanger sequencing and Infinium Global Screening Array by Macrogen Inc. (Seoul, Korea).

### 4.7. Annotation

The ‘golden set’ was annotated using gene-based, region-based, and filter-based annotation provided by Annovar. RefSeq database was used for annotating variants overlapping with the coding regions and for the non-coding elements, wgRNA and GENCODE (https://www.gencodegenes.org (accessed on 15 November 2020)) databases were utilized. TCAG annotation pipeline (rev26.6) was used to annotate the SRS set variants in VCF format and used Annovar software to functionally annotate the variants.

### 4.8. Plots and Graphs

PycoQC (https://github.com/a-slide/pycoQC (accessed on 15 November 2020)), MultiQC (https://github.com/ewels/MultiQC (accessed on 15 November 2020)), and Circos software (http://circos.ca (accessed on 15 November 2020)) were used to achieve base-called and aligned statistics and plots.

## Data Availability

Data is contained within the supplementary material. The data presented in this study is available in Golden_Set.hg38_multianno.txt.

## Supplementary Materials

The following can be found online at https://www.mdpi.com/1422-0067/22/4/2060/s1, Table S1: Summary of sequencing and alignment statistics of all samples, Figure S1: Pedigree and Sanger validation of CLCA4 and DXO mutation in the family, Figure S2: A density plot of the basecalled read length compared with the mean read PHRED quality scores.

## Abbreviations

WGS	Whole-Genome Sequencing
ONT	Oxford Nanopore Technology
NcRNA	Non-coding RNA
MZ	Monozygotic Twins
RO	Reciprocal Overlap
