# Supplementary material for: Long-Read Sequencing Improves the Detection of Structural Variations Impacting Complex Non-Coding Elements of the Genome
Source: Int J Mol Sci. 2021 Feb 19;22(4):2060. doi: 10.3390/ijms22042060 (PMC7923155; doi:10.3390/ijms22042060)
Supplement: Supplementary file 1 [file ijms-22-02060-s001.zip › Supplementary_Figures.docx]

Long read sequencing improves detection of structural variations within complex non-coding regions of the genome

1. Supplementary Tables and Figures

**Table S1.** Summary of sequencing and alignment statistics of all samples


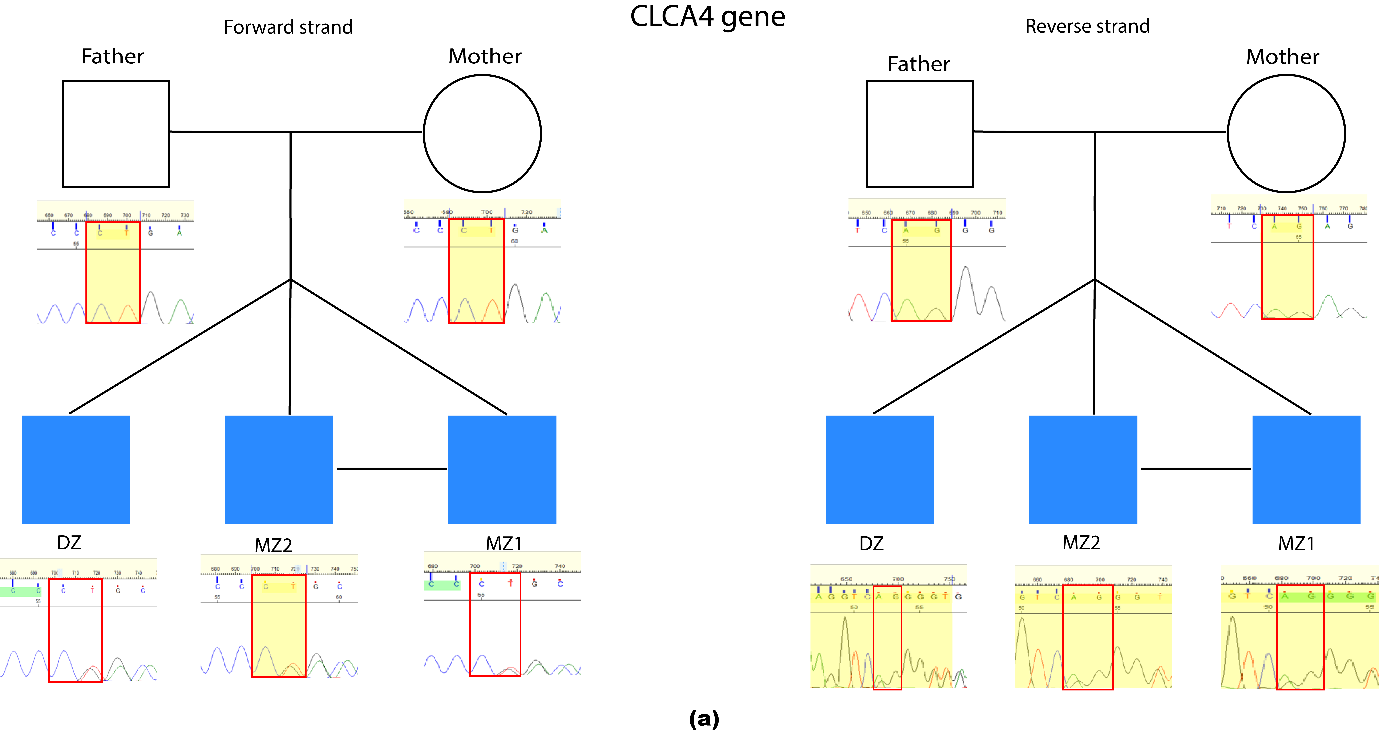


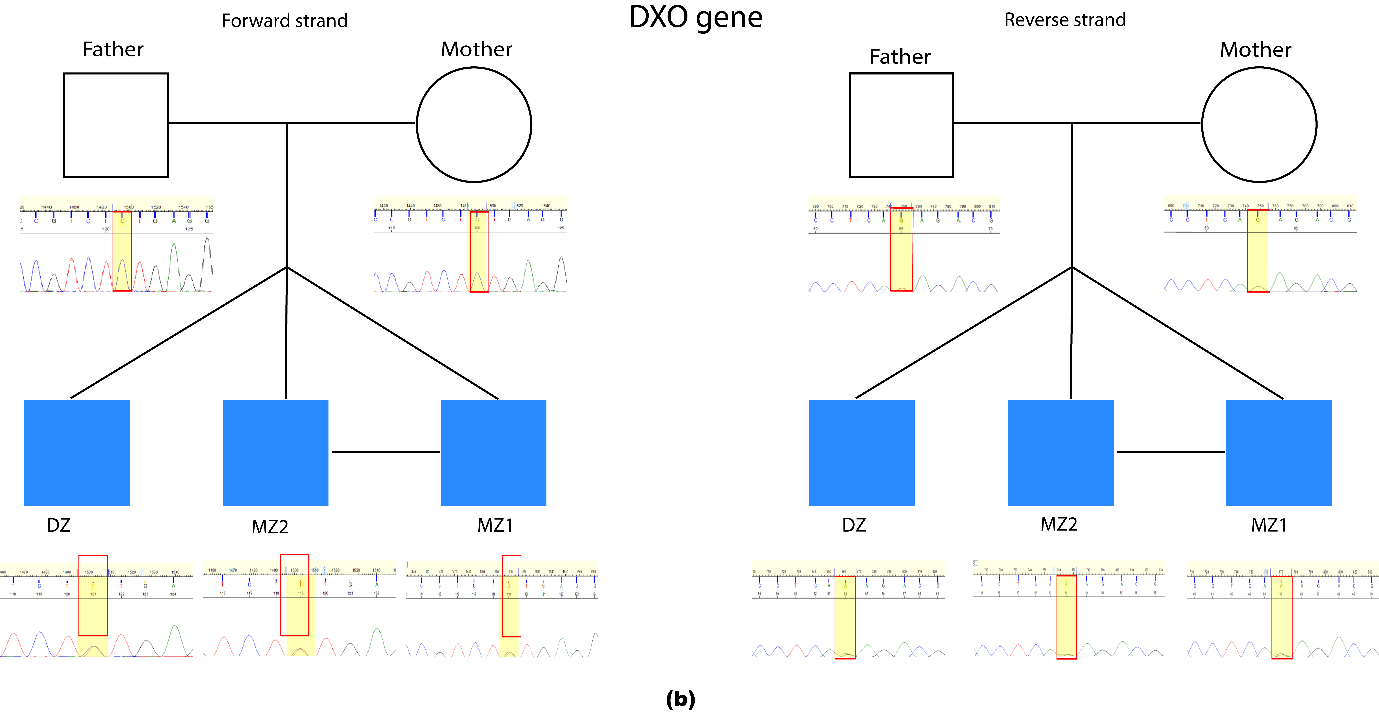


**Figure S1**. Pedigree and Sanger validation of CLCA4 and DXO mutation in the family: (**a**) Sanger validation of 1 bp deleted variant (c.87025997_87025998delT) in the CLCA4 gene. The sequence chromatograph showed a double peak on the affected triplets (DZ, MZ1 and MZ2) and single peak on the parents confirm that it is a heterozygous deletion. (**b**) Sanger validation of g.31939199:C>T in only the affected triplet’s siblings, confirmed by both the forward and reverse strand while the unaffected parents sample contained the reference allele thus confirming the *de novo* SNP mutation.


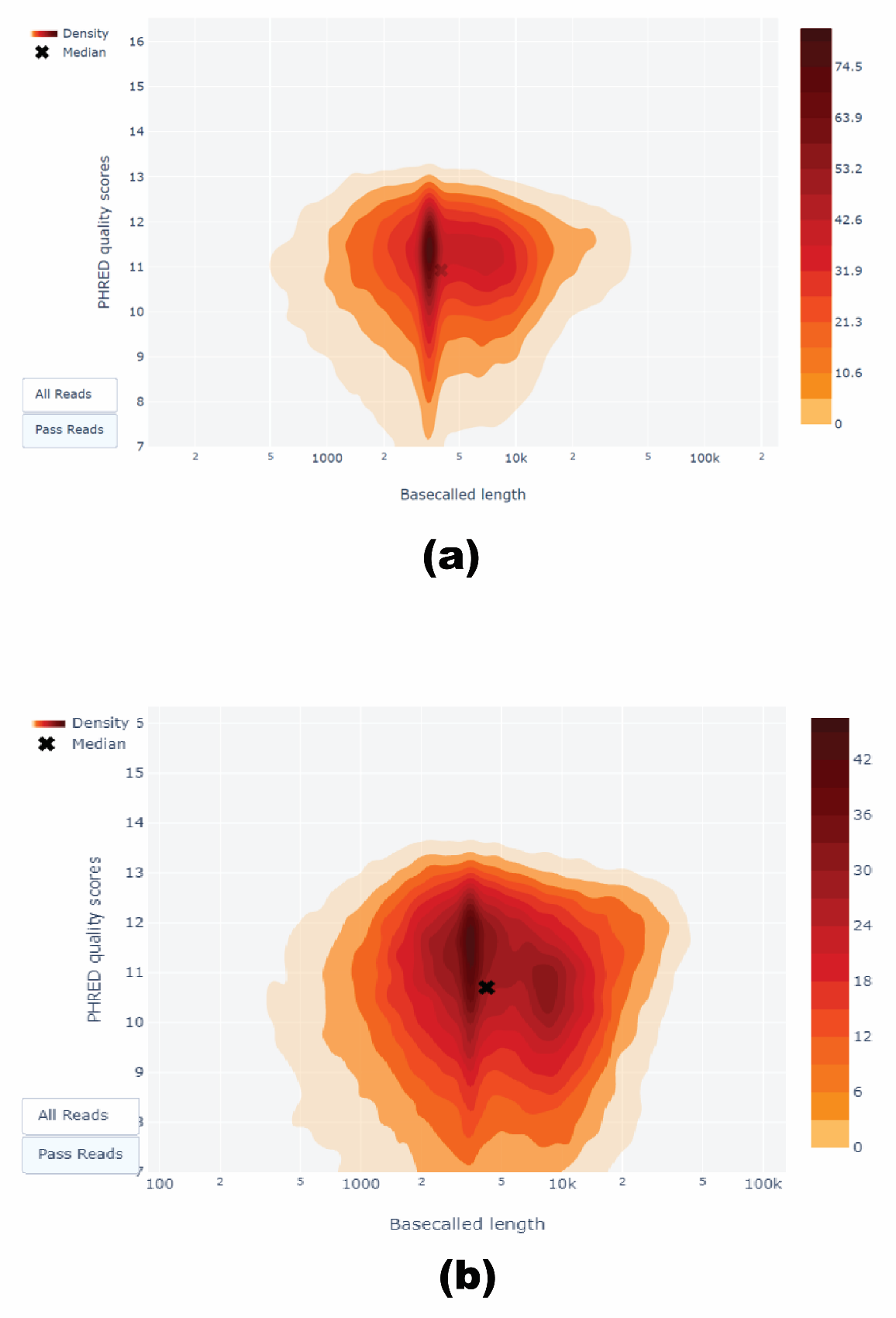


**Figure S2**. A density plot of the basecalled read length compared with the mean read PHRED quality scores. (**a**) The 2D plot is generated for monozygotic twin 1 (MU006 sample). (**b**) The 2D plot is generated for monozygotic twin 2 (MU007). The x denotes the median and the plot was generated using pycoQC.


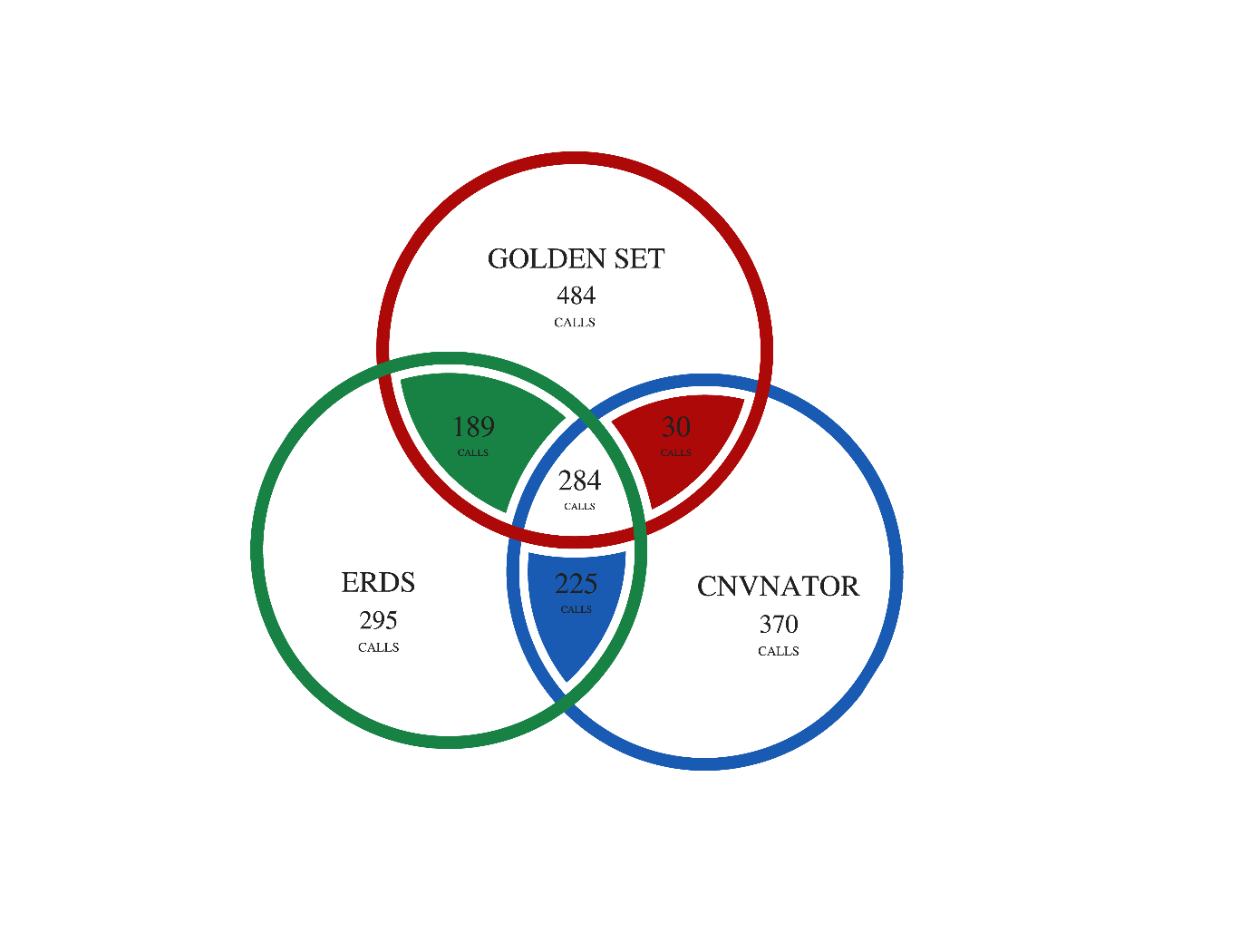


**Figure S3**. Venn diagram representing unique and shared variants between both platforms (Illumina and nanopore), after applying a reciprocal overlap threshold of 50%. The overlap was performed between the following sets: illumina callers set of ERDS and CNVNATOR (in blue), ‘golden set’ and ERDS set (in green), ‘golden set’ and CNVnator set (in red), and ‘golden set’ and illumina consensus set (in white), which resulted in 225, 473, 314, and 284 SV calls overlapping between sets, respectively. The numbers below each labelled set represents unique variants to that particular set.
