# Supplementary figures and images for: Long-Read Sequencing Improves the Detection of Structural Variations Impacting Complex Non-Coding Elements of the Genome
Source: Int J Mol Sci. 2021 Feb 19;22(4):2060. doi: 10.3390/ijms22042060 (PMC7923155; doi:10.3390/ijms22042060)

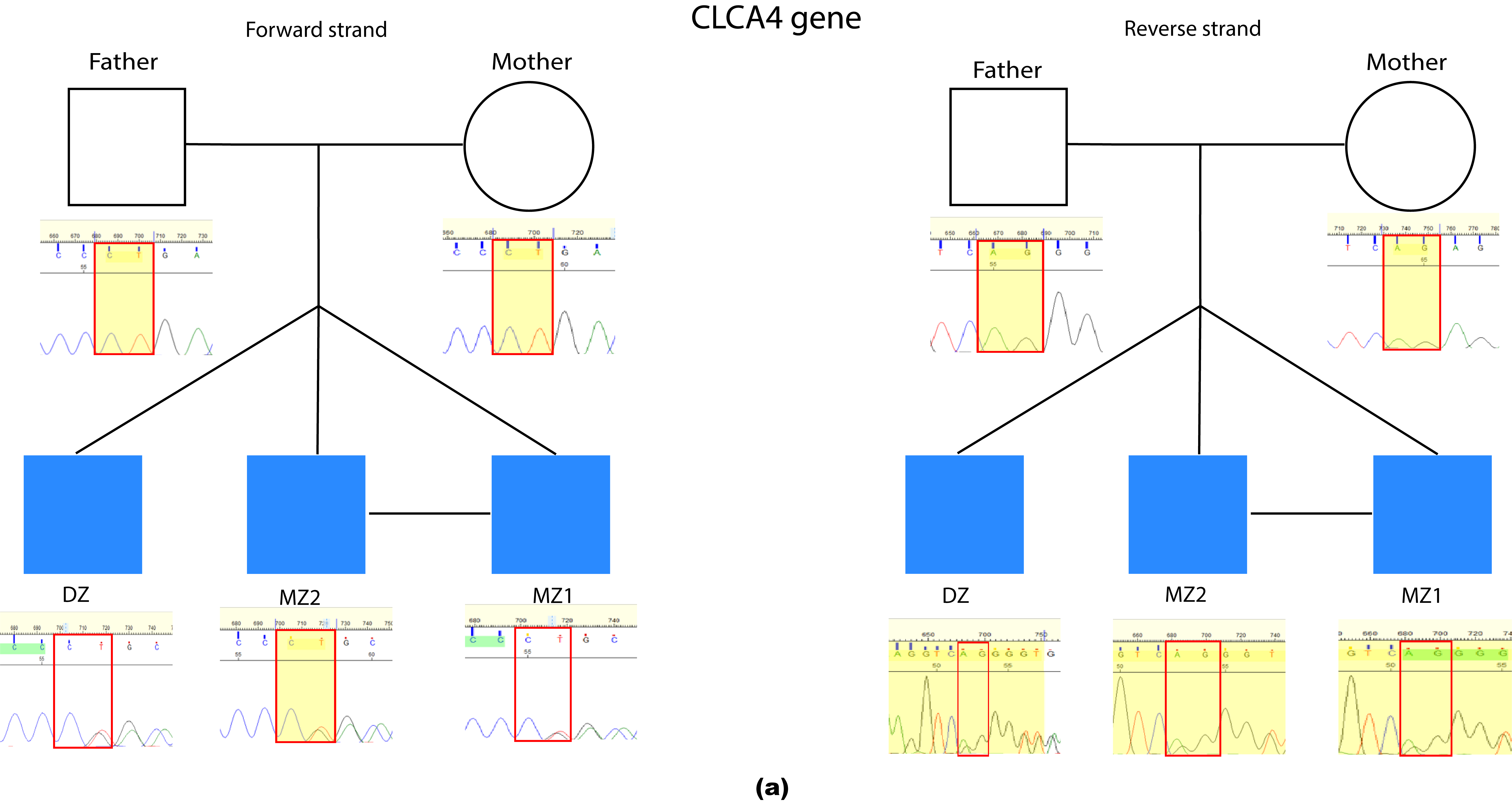

Supplement: Supplementary file 1 [file ijms-22-02060-s001.zip › Figure S1a.tif]

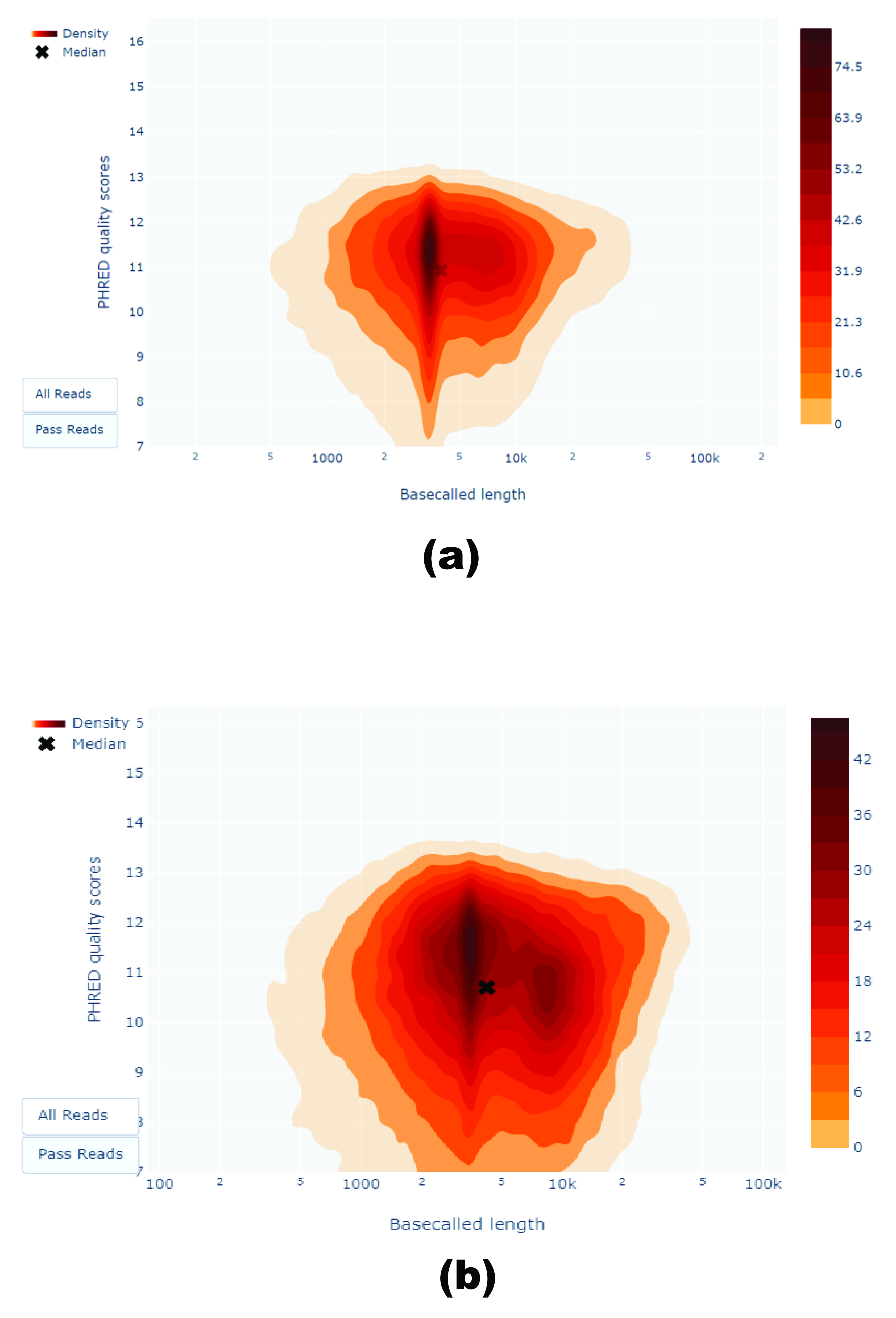

Supplement: Supplementary file 1 [file ijms-22-02060-s001.zip › Figure S2.tif]

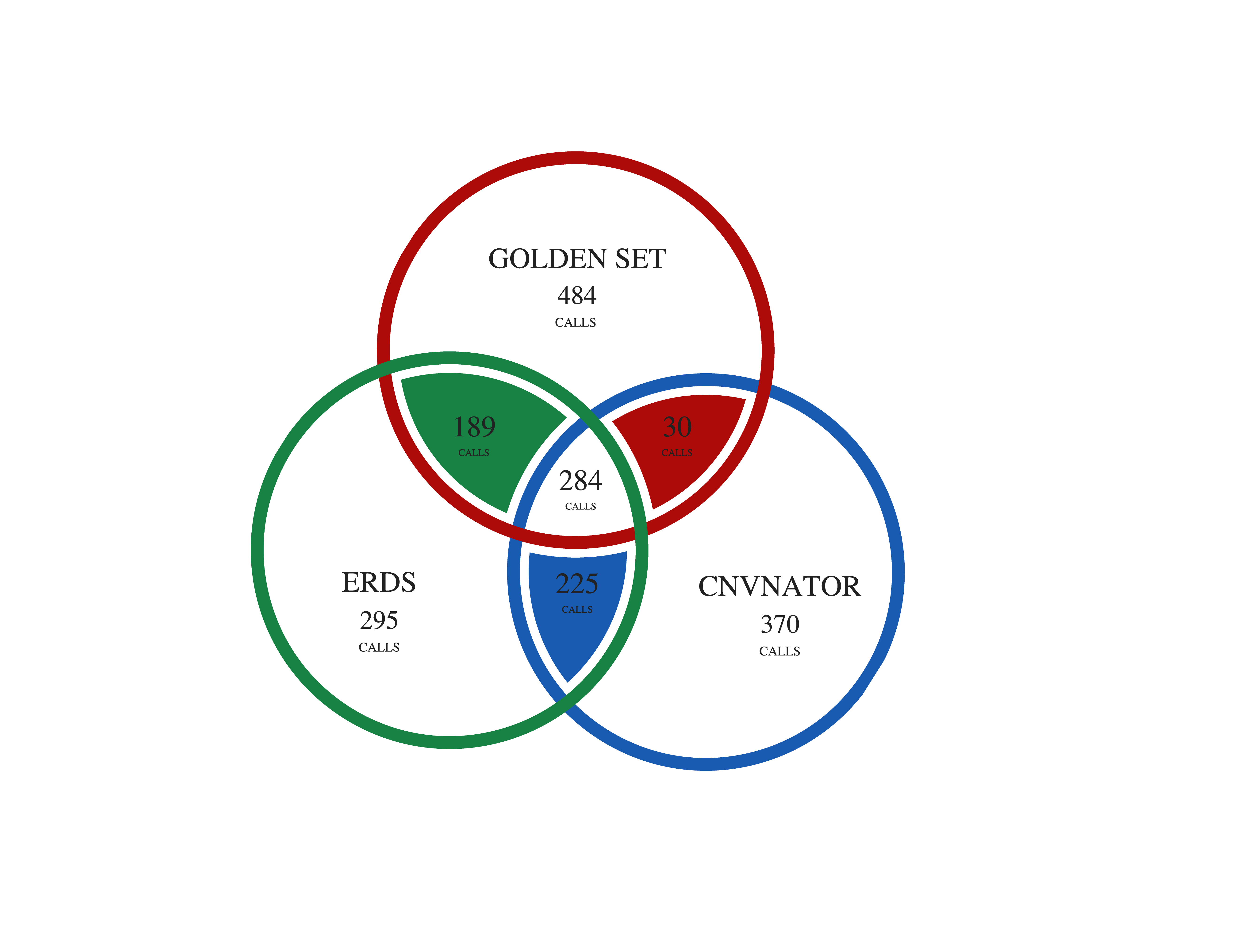

Supplement: Supplementary file 1 [file ijms-22-02060-s001.zip › Figures S3.tif]

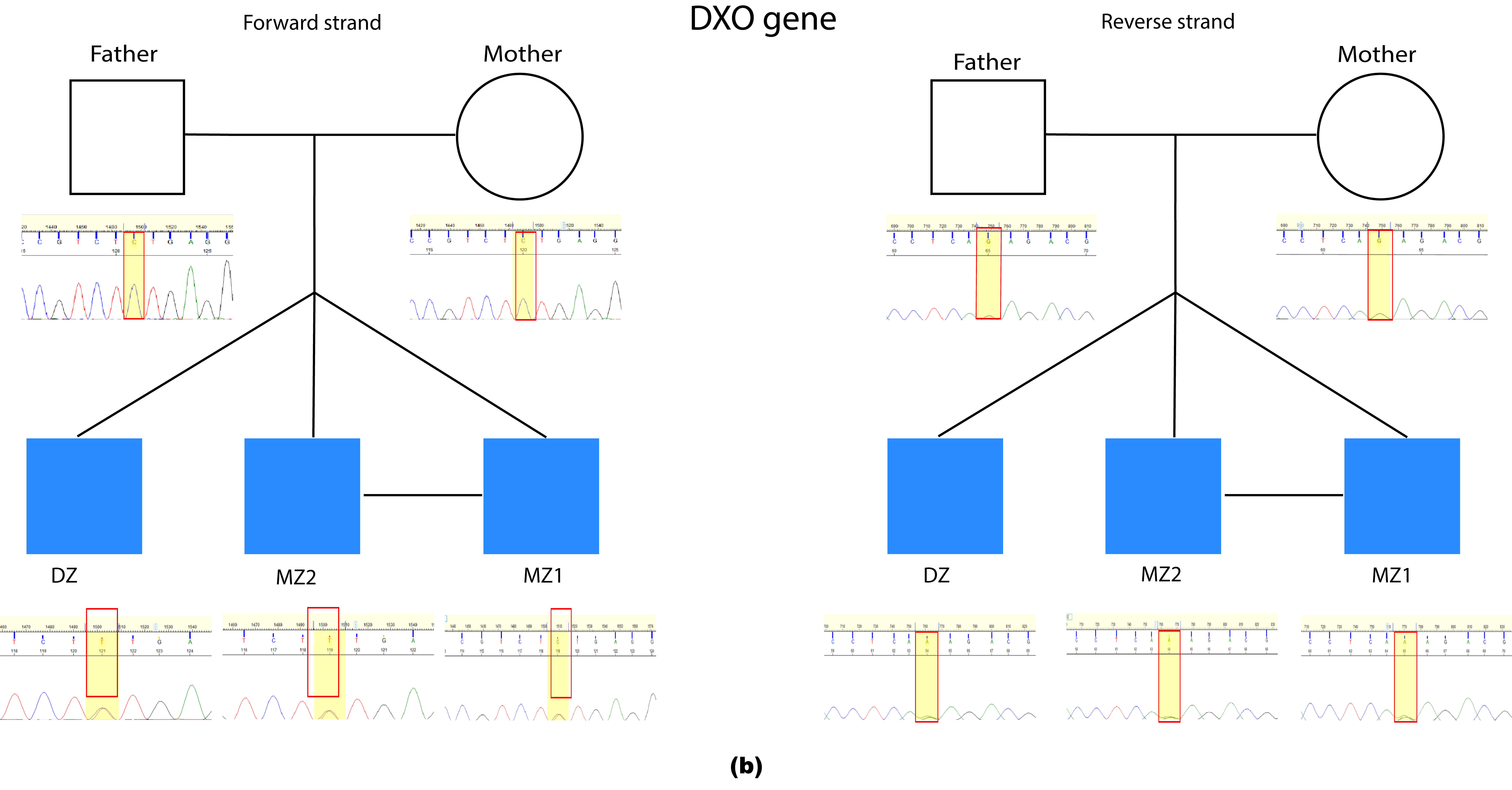

Supplement: Supplementary file 1 [file ijms-22-02060-s001.zip › Figure S1b.tif]
